# Long-Term Real-World Experience with Safinamide in Patients with Parkinson’s Disease

**DOI:** 10.3390/brainsci14121238

**Published:** 2024-12-09

**Authors:** Anna Planas-Ballvé, Núria Caballol Pons, Alejandro Peral Quirós, Isabel Gómez Ruiz, Marta Balagué Marmaña, Alexander J. Velázquez Ballester, Dolors Lozano Moreno, Asunción Ávila Rivera

**Affiliations:** Movement Disorders Unit, Neurology Department, Complex Hospitalari Moisès Broggi, 08970 Sant Joan Despí, Barcelona, Spain; annaplanasb@gmail.com (A.P.-B.); caballol@csi.cat (N.C.P.); aperalq@csi.cat (A.P.Q.); migomez@csi.cat (I.G.R.); mbalague@csi.cat (M.B.M.); ajvelazquezb@csi.cat (A.J.V.B.); dlozano@csi.cat (D.L.M.)

**Keywords:** Parkinson’s disease, safinamide, MAO-B inhibitor, real-world evidence, discontinuation rate, motor symptoms

## Abstract

Introduction: Randomized clinical trials should be complemented with data from real-world studies. We report our long-term experience with safinamide in a movement disorders unit. Methods: This retrospective study included patients with Parkinson’s disease (PD) treated with safinamide in our unit from February 2016 to May 2022 under routine clinical practice. Assessments included the Hoehn and Yahr (HY) stage, unified Parkinson’s disease rating scale (UPDRS) part III score, levodopa equivalent daily dose (LEDD), LEDD for dopamine agonists, and safinamide treatment discontinuation. Results: We included 180 patients with a median age of 74 years (IQR 11), and the majority (90.6%) had an HY stage of ≤2. After a median follow-up of 40 months (IQR 34), 14 patients discontinued treatment with safinamide (7.8%, 95% CI 4.7 to 12.6). Among the 166 patients who remained on safinamide, the UPDRS III score was stable (10 (IQR 9) vs. 9 (IQR 13), *p* = 0.455). The LEDD significantly increased from a median of 300 mg to 500 mg (*p* < 0.001), whereas the LEDD for dopamine agonists did not significantly increase. A subgroup of 89 patients who did not require dopamine agonists during follow-up showed stable UPDRS III score (10 (IQR 7) vs. 9 (IQR 14); *p* = 0.923), with a significant LEDD increase (300 mg to 400 mg, *p* < 0.001). Conclusions: Our results support the long-term effectiveness and tolerability of safinamide in patients with PD in clinical practice.

## 1. Introduction

Parkinson’s disease (PD) is a progressive neurodegenerative disorder that significantly affects motor and non-motor functions, leading to disability and impaired quality of life [1]. The hallmark motor symptoms include bradykinesia, rigidity, and tremor, which are caused by the degeneration of dopaminergic neurons in the substantia nigra [2]. In addition to motor symptoms, PD is associated with a wide range of non-motor manifestations, such as mood disorders, cognitive impairment, sleep disturbances, and pain, which further contribute to the disease burden [3,4].

Levodopa is the cornerstone treatment for PD [5]. However, despite its efficacy in the management of PD, the use of levodopa is associated with motor complications, such as motor fluctuations and dyskinesias. A review of the literature revealed that after 4–6 years of treatment with levodopa, approximately 40% of patients develop dyskinesias, and 40% develop motor fluctuations [6]. These motor complications compromise the efficacy of treatment and are associated with disability and impaired quality of life [7,8].

In addition to optimizing levodopa treatment, the management of motor complications associated with the use of levodopa includes adjunctive therapies such as dopamine agonists, catechol-O-methyltransferase (COMT) inhibitors, and monoamine oxidase B (MAO-B) inhibitors [9]. While experts agree that none of these options are clearly superior in terms of efficacy and tolerability [10], a recent pragmatic randomized clinical trial comparing these three therapies in patients with PD and levodopa-related motor complications found no functional advantage of dopamine agonists over the combined group of MAO-B or COMT inhibitors, as evaluated with the mobility subscale of the 39-item Parkinson’s disease questionnaire (PDQ-39). Interestingly, the MAO-B group showed significantly better mobility scores and EuroQol 5-dimensional 3-level utility scores compared to the COMT inhibitor group [11]. Based on these findings, the authors of the trial suggested that MAO-B inhibitors may be underused in clinical practice.

Safinamide is a highly selective and potent MAO-B inhibitor that modulates both the dopaminergic and glutamatergic systems [12]. Several randomized clinical trials have demonstrated the efficacy of safinamide in the treatment of motor symptoms in patients with PD [13,14,15,16,17]. In addition, safinamide has a positive effect on non-motor symptoms, such as pain [18], mood disorders [19,20], cognitive function [21,22], sleep disorders [23,24], and urinary symptoms [25]. Randomized clinical trials (RCTs) are considered the gold standard for evaluating the effect and value of an intervention [26]. However, populations included in clinical trials conducted in patients with PD differ from those treated in clinical practice in terms of relevant characteristics, such as time to diagnosis or treatment initiation, which are longer in real-world populations [27]. Therefore, as has been claimed, information from RCTs should be complemented with data from real-world observational studies. Treatment outcomes with safinamide under real-life conditions have been reported in several studies, most of which involved a small cohort of patients and/or short-term follow-up [25,28,29,30,31,32,33,34,35]. In the largest observational study conducted so far, the SYNAPSES trial, patients treated with safinamide were followed for up to 1 year [36]. Here, we report our long-term experience with safinamide in a movement disorders unit.

The current study aims to evaluate the long-term effectiveness and safety of safinamide in real-world clinical settings, providing valuable insights into its impact on patients with PD in routine clinical practice. Here, we report our long-term experience with safinamide in our movement disorders unit, contributing to the growing body of evidence supporting its use. Our findings seek to bridge the gap between clinical trial data and real-life experiences, highlighting the practical benefits of safinamide in managing PD.

## 2. Materials and Methods

### 2.1. Study Design

This was a retrospective, observational study conducted at the Movement Disorders Unit of the Complex Hospitalari Moisès Broggi (Barcelona, Spain). The study was approved by the Ethics Committee of our center (Reference PR211/22 [CSI22/56]), and written informed consent was obtained from all participants.

### 2.2. Participants

We included all patients diagnosed with PD who were treated with safinamide in our unit between February 2016 and May 2022. No additional selection criteria were applied. However, most patients with late-stage PD requiring advanced therapies (such as duodenal levodopa/carbidopa infusion therapy or deep brain stimulation) were treated at tertiary referral hospitals outside our unit. Subgroup analyses were performed to evaluate specific patient groups: patients who did not require adjunctive therapy with dopamine agonists during follow-up, patients who were not receiving levodopa at baseline or during the follow-up period, and patients treated with safinamide in monotherapy and in combination with dopamine agonists but without levodopa.

### 2.3. Procedures and Instruments

At treatment initiation, we collected baseline data, including age, time since the onset of PD symptoms, and time from PD diagnosis. During follow-up visits, clinical assessments included the Hoehn and Yahr (HY) stage to evaluate disease stage and the unified Parkinson’s disease rating scale (UPDRS) part III score (evaluated during the On state) to assess motor impairment. The levodopa equivalent daily dose (LEDD) and LEDD for dopamine agonists was calculated using standardized conversion formulas. Additionally, safinamide treatment discontinuation was documented, along with the reasons for discontinuation, such as side effects or lack of efficacy.

### 2.4. Data Collection and Analysis

The study data were collected and managed using research electronic data capture (REDCap), a secure web-based software platform that facilitates data collection, audit trails, data export, and integration with external sources (https://projectredcap.org) [37,38]. Continuous variables are presented as medians and interquartile ranges (IQRs), while categorical variables are reported as absolute and relative frequencies. The percentage and 95% confidence interval (CI) for safinamide treatment discontinuation were calculated. Quantitative outcomes at treatment initiation and the last follow-up visit were compared using the Wilcoxon signed-rank test for paired data. For comparisons between independent groups, the Mann–Whitney U test was used. Categorical outcomes were analyzed with the chi-square test. All tests were two-sided and considered statistically significant at *p* < 0.05. Statistical analyses were performed using IBM SPSS version 26 software.

## 3. Results

During the study period, a total of 180 patients with PD were treated with safinamide in our unit. The median age of the patients was 74 years (IQR 11), and the majority (90.6%) were in HY stage ≤ 2. Baseline demographic and clinical characteristics are summarized in Table 1.

After a median follow-up period of 40 months (IQR 34), only 14 patients discontinued treatment with safinamide (7.8%, 95% CI 4.7 to 12.6). The reasons for discontinuation included side effects (*n* = 6), lack of efficacy (*n* = 6), and unknown reasons (*n* = 2). The most frequently reported side effects leading to discontinuation were dizziness and vomiting (*n* = 3), followed by nervousness (*n* = 1), constipation (*n* = 1), and visual hallucinations (*n* = 1).

Among the 166 patients who continued treatment with safinamide, motor function as measured by the UPDRS III score remained stable between baseline and the final follow-up visit (10 (9) vs. 9 (13); *p* = 0.455). However, the LEDD increased significantly from a median of 300 mg to 500 mg (*p* < 0.001), while the LEDD for dopamine agonists did not show significant changes (*p* = 0.531). These results are detailed in Table 2.

A subgroup analysis was performed for 89 patients who did not require adjunctive dopamine agonists during treatment. In this group, the UPDRS III score remained stable (10 (7) vs. 9 (14); *p* = 0.923), while the LEDD increased significantly from a median of 300 mg to 400 mg (*p* < 0.001). Results for this subgroup are also shown in Table 2.

Another subgroup consisted of 23 patients who were not receiving levodopa at baseline or throughout the follow-up period. Of these, 18 were treated with safinamide monotherapy, and 13 remained on monotherapy throughout the follow-up period, which had a median duration of 28 months (IQR 37). In this subgroup, there was a significant improvement in motor function, as the UPDRS III score decreased from 5 (3) to 4 (2) (*p* = 0.006), and all patients maintained HY stage ≤ 2 throughout the study.

At the final follow-up, 6 patients were treated with safinamide in combination with dopamine agonists. Among them, 3 patients were already on dopamine agonists before starting safinamide, and they showed an improvement in UPDRS III scores (median decreased from 5 to 4). The remaining 3 patients initiated dopamine agonists during follow-up, and their UPDRS III scores remained stable (median of 5).

## 4. Discussion

This study evaluated the long-term effectiveness and tolerability of safinamide in real-world conditions, with a follow-up of over three years. The key findings were a low treatment discontinuation rate, stability in motor symptoms, and a modest increase in the LEDD, with no significant changes in the LEDD for dopamine agonists. These results support the role of safinamide as a valuable therapeutic option for patients with PD, particularly in earlier stages with mild-to-moderate symptoms.

The discontinuation rate of safinamide after more than three years (7.8%) was notably lower than that of other studies involving safinamide or other anti-Parkinson’s drugs in both RCTs and real-world settings. For example, in an RCT with patients with early PD, Stocchi et al. reported a 10% discontinuation rate after 24 weeks and 20% after one year [16]. Similarly, another trial in patients with PD and motor fluctuations reported a discontinuation rate of 10.6% after 24 weeks of safinamide treatment [17]. Importantly, the SYNAPSES trial, the largest observational study on safinamide to date, reported a 21.6% discontinuation rate after one year [36]. In contrast, the PD MED pragmatic trial revealed a much higher discontinuation rate (over 40%) for various add-on treatments, such as dopamine agonists, MAO-B inhibitors, or COMT inhibitors [11]. Comparable discontinuation rates have been observed in other real-world studies; for instance, rasagiline had a 21.2% discontinuation rate owing to adverse events [39], and opicapone exhibited a 20.6% discontinuation rate within three months [40]. Our study’s low discontinuation rate of 4% due to adverse events, compared to 10% in the SYNAPSES trial, may reflect differences in patient profiles, as our cohort included patients in earlier disease stages. These findings suggest that safinamide might be particularly effective when introduced early in the treatment pathway for patients exhibiting mild to moderate symptoms and minimal to non-troublesome dyskinesia, which is consistent with expert opinions on the ideal patient profile for safinamide treatment [41]. Some neurologists have also used safinamide in patients with barely perceptible fluctuations or in those reporting suboptimal clinical status throughout the day. Further research is needed to substantiate these observations, although they are somewhat supported by expert consensus [41].

Our findings on the discontinuation rate are further reinforced by the results of other outcomes, such as the progression of the UPDRS III (motor) score and LEDD. Over the 3-year treatment period with safinamide, the UPDRS III score remained stable, highlighting its long-term effectiveness. This contrasts with findings from Schrag et al., who reported a median increase of 3.3 points in the UPDRS III score over 1 year in a clinic-based sample of 128 patients with PD [42]. Notably, patients with PD in real-world settings tend to experience a faster progression of motor symptoms compared to those in clinical trials [27]. In our cohort, the LEDD showed a modest but statistically significant increase during follow-up, while the LEDD for dopamine agonists remained stable. Interestingly, we previously reported that rasagiline, under clinical practice conditions, was associated with an increase in the LEDD for dopamine agonists [43]. This stability in dopamine agonist use observed with safinamide may have contributed to the favorable long-term tolerability observed in our study. Dopamine agonists are known to carry a higher overall risk of side effects compared to levodopa, including psychosis, excessive daytime sleepiness, peripheral edema, and impulse control disorders [44].

Our results regarding the use of safinamide monotherapy in a small subset of patients are encouraging, with a significant improvement in motor symptoms after 3 years of treatment. Rasagiline has been demonstrated to be efficacious as a monotherapy for the treatment of early PD in randomized clinical trials [45,46]. However, we are not aware of any randomized clinical trials that have evaluated safinamide in this clinical setting. In a subgroup analysis of the 009 trial with safinamide, the administration of a 1.0 mg/kg daily dose was superior in terms of the UPDRS-III response rate (i.e., at least a 30% reduction in the UPDRS-III score) at 12 weeks [47]. The potential role of MAO-B inhibitors as monotherapy for patients with PD and minor disabilities has been recognized by experts [5]. Therefore, randomized controlled trials of safinamide in this population are needed.

This study has some limitations that should be acknowledged. First, the cohort included mostly patients in the early stages of PD. This limits the generalizability of the findings to more advanced stages, where motor complications are more pronounced, and treatment is more challenging. Second, the study lacked a control group of patients either not treated with MAO-B inhibitors or treated with alternative therapies, such as rasagiline. This limits the ability to directly compare safinamide’s effectiveness and tolerability with other options. Third, while information on side effects leading to treatment discontinuation was collected, data on side effects that did not result in discontinuation were not systematically gathered, which limits the overall assessment of tolerability. Finally, non-motor symptoms and patient-reported outcomes were not evaluated, which could have provided a more comprehensive assessment. Despite these limitations, the study offers valuable insights into the real-world long-term use of safinamide in patients with mild to moderate PD.

Further research is warranted to explore the use of safinamide as monotherapy, particularly in patients with early PD and minor disabilities. While our findings in this subset of patients are promising, randomized controlled trials are needed to confirm its efficacy in this setting. Additionally, future studies should include non-motor symptoms and patient-reported outcomes to provide a more comprehensive evaluation of the drug’s impact.

In conclusion, overall, our findings support the long-term effectiveness and tolerability of safinamide in clinical practice. This study underscores its potential as an important treatment option for patients with PD, particularly when introduced early in the disease course.

## Figures and Tables

**Table 1:** Demographic and clinical characteristics of patients treated with safinamide.

Characteristic	*n* = 180
Age, Median (IQR)	74 Years (11)
Time from PD onset of symptoms, median (IQR)	43.5 months (61)
Time from PD diagnosis, median (IQR)	25 months (63)
HY stage ≤ 2, *n*, (%)	163 (90.6%)
UPDRS III score, median (IQR)	10 (12)
LEDD, median (IQR)	300 mg (559)
LEDD of dopamine agonists, median (IQR)	0 mg (148)

**Table 2:** Outcome measures after long-term treatment with safinamide.

	Treatment Initiation	Final Follow-Up Visit	*p* Value
All patients who were on safinamide at the end of the follow-up (*n* = 166)			
UPDRS III score, median (IQR)	10 (9)	9 (13)	0.455
HY stage ≤ 2, *n*, (%)	151 (91)	136 (82)	0.006
LEDD, median (IQR)	300 mg (564)	500 mg (431)	<0.001
LEDD of dopamine agonists, median (IQR)	0 mg (158)	0 mg (160)	0.531
Patients who were on safinamide at the end of the follow-up and did not require dopamine agonists (*n* = 89)			
UPDRS III score, median (IQR)	10 (7)	9 (14)	0.923
HY stage ≤ 2, *n*, (%)	82 (92.1)	73 (82)	0.049
LEDD, median (IQR)	300 mg (375)	400 mg (325)	<0.001

## Data Availability

The data that support the findings of this study are available from the corresponding author upon reasonable request. The data are not publicly available due to privacy reasons.
